# *catena*-Poly[[(1,10-phenanthroline-κ^2^ *N*,*N*′)lead(II)]-di-μ-nitrato-κ^3^ *O*,*O*′:*O*′′;κ^3^ *O*:*O*′,*O*′′-[(1,10-phenanthroline-κ^2^ *N*,*N*′)lead(II)]-bis­(μ-2,2,2-trichloro­acetato-κ^2^ *O*:*O*′)]

**DOI:** 10.1107/S1600536812003595

**Published:** 2012-02-04

**Authors:** Gholam Hossein Shahverdizadeh, Seik Weng Ng, Edward R. T. Tiekink, Babak Mirtamizdoust

**Affiliations:** a Department of Chemistry, Faculty of Science, Tabriz Branch, Islamic Azad University, PO Box 1655, Tabriz, Iran; b Department of Chemistry, University of Malaya, 50603 Kuala Lumpur, Malaysia; c Chemistry Department, Faculty of Science, King Abdulaziz University, PO Box 80203 Jeddah, Saudi Arabia; d Department of Inorganic Chemistry, Faculty of Chemistry, University of Tabriz, PO Box 5166616471, Tabriz, Iran

## Abstract

In the title Pb^II^ complex, [Pb_2_(C_2_Cl_3_O_2_)_2_(NO_3_)_2_(C_12_H_8_N_2_)_2_]_*n*_, the 1,10-phenanthroline ligand is chelating, the nitrate anion chelates one Pb^II^ ion and simultaneously bridges a neighbouring Pb^II^ ion *via* the third O atom, and the trichloro­acetate anion is bidentate, bridging two Pb^II^ ions. The coordination geometry is based on a penta­gonal–bipramidal geometry defined by an N_2_O_5_ donor set with no obvious stereochemical role for the lead-bound lone pair of electrons. The coordination polymer has a zigzag topology along [010] and comprises alternating eight-membered {PbONO}_2_ and {PbOCO}_2_ rings.

## Related literature

On the stereochemical activity of lone pairs of electrons in Pb^II^ structures, see: Davidovich *et al.* (2009 ▶). For recent structural studies of mixed-ligand Pb^II^ compounds, see: Shahverdizadeh *et al.* (2008 ▶, 2011*a* ▶,*b* ▶). For specialized crystallization techniques, see: Harrowfield *et al.* (1996 ▶).
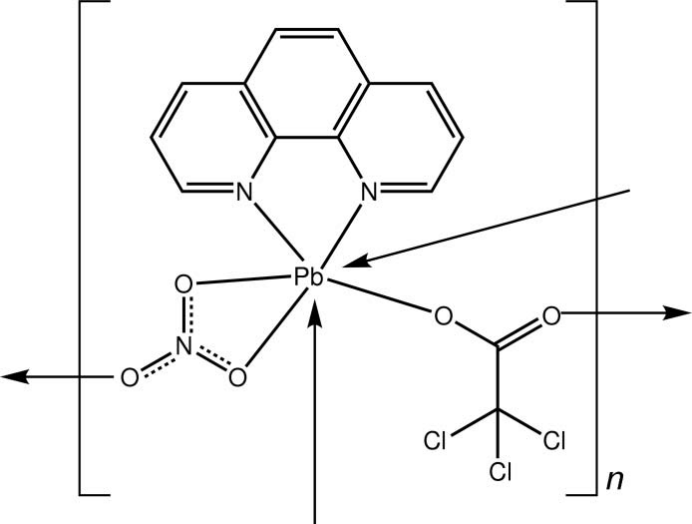


## Experimental

### 

#### Crystal data

[Pb_2_(C_2_Cl_3_O_2_)_2_(NO_3_)_2_(C_12_H_8_N_2_)_2_]
*M* *_r_* = 1223.54
Triclinic, 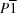

*a* = 8.8852 (3) Å
*b* = 9.7465 (3) Å
*c* = 11.1570 (3) Å
α = 109.427 (3)°
β = 99.019 (3)°
γ = 106.199 (3)°
*V* = 841.13 (4) Å^3^
*Z* = 1
Mo *K*α radiation
μ = 10.54 mm^−1^
*T* = 100 K
0.20 × 0.15 × 0.10 mm

#### Data collection

Agilent SuperNova Dual diffractometer with an Atlas detector
Absorption correction: multi-scan (*CrysAlis PRO*; Agilent, 2010 ▶) *T*_min_ = 0.227, *T*_max_ = 0.419
12671 measured reflections
3899 independent reflections
3740 reflections with *I* > 2σ(*I*)
*R*_int_ = 0.029

#### Refinement

*R*[*F* ^2^ > 2σ(*F* ^2^)] = 0.017
*wR*(*F* ^2^) = 0.038
*S* = 0.99
3899 reflections
235 parameters
H-atom parameters constrained
Δρ_max_ = 0.84 e Å^−3^
Δρ_min_ = −0.97 e Å^−3^

### 

Data collection: *CrysAlis PRO* (Agilent, 2010 ▶); cell refinement: *CrysAlis PRO*; data reduction: *CrysAlis PRO*; program(s) used to solve structure: *SHELXS97* (Sheldrick, 2008 ▶); program(s) used to refine structure: *SHELXL97* (Sheldrick, 2008 ▶); molecular graphics: *X-SEED* (Barbour, 2001 ▶) and *DIAMOND* (Brandenburg, 2006 ▶); software used to prepare material for publication: *publCIF* (Westrip, 2010 ▶).

## Supplementary Material

Crystal structure: contains datablock(s) global, I. DOI: 10.1107/S1600536812003595/hg5168sup1.cif

Structure factors: contains datablock(s) I. DOI: 10.1107/S1600536812003595/hg5168Isup2.hkl

Additional supplementary materials:  crystallographic information; 3D view; checkCIF report

## Figures and Tables

**Table 1:** Selected bond lengths (Å)

Pb—O1	2.410 (2)
Pb—O2^i^	2.821 (2)
Pb—O3	2.591 (2)
Pb—O4	2.844 (2)
Pb—O5^ii^	2.807 (2)
Pb—N1	2.576 (2)
Pb—N2	2.515 (2)

Symmetry codes: (i) 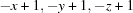; (ii) 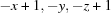.

**Fig. 1.**
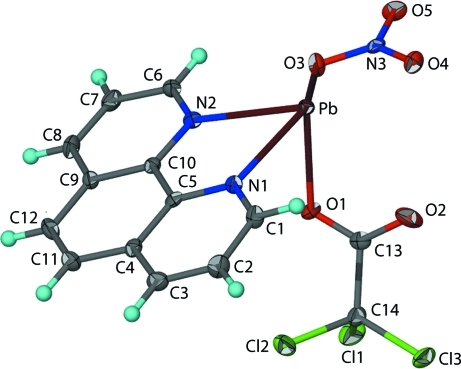
The asymmetric unit of (I) showing the atom-labelling scheme and displacement ellipsoids at the 70% probability level.

**Fig. 2.**
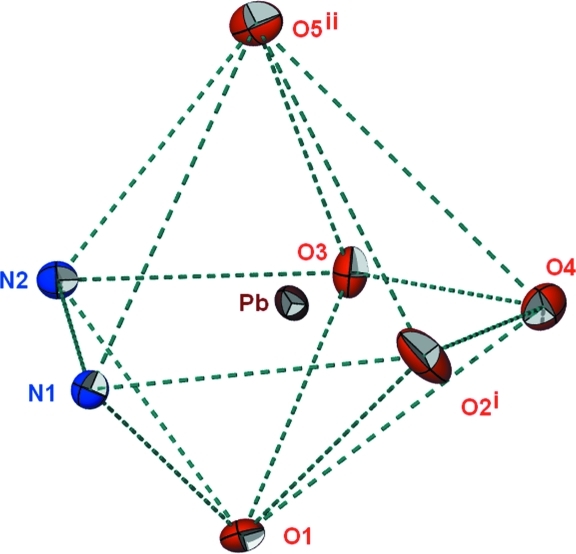
A view of the distorted pentagonal bipyramidal coordination geometry for the lead atom in (I).

**Fig. 3.**
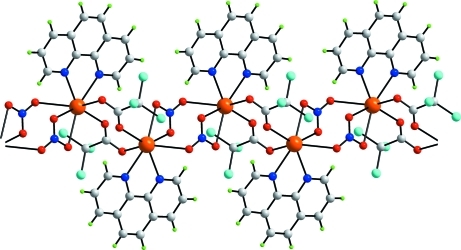
A view of the zigzag polymeric chain along [010] in (I).

## supplementary crystallographic information

### Comment

The coordination chemistry of Pb^II^ with N– and O-donor ligands has been investigated in the past decade and frequently discussed in regard of the stereochemical activity of the lone pair of electrons (Davidovich *et al.*, 2009). In connection with recent structural studies (Shahverdizadeh *et al.*, 2008, 2011*a*, 2011*b*), herein we report the cyrstal and molecular structure of the mixed ligand Pb^II^ complex containing nitrate, trichloroacetate and 1,10-phenanthroline, (I).

A view of the asymmetic unit is shown in Fig. 1. This comprises a Pb^II^ atom, a chelating 1,10-phenanthroline molecule, a nitrate anion and a trichloroacetate anion. The nitrate anion chelates one Pb^II^ atom and at the same time bridges a second Pb^II^ atom *via* the third O atom. The trichloroacetate anion is bidentate, bridging two Pb^II^ atoms. Both bridges are non-symmetric, Table 1. The resulting coordination geometry, Fig. 2, is based on a distorted pentagonal bipyramid with no obvious role for the lead-bound lone pair of electrons.

The resulting polymeric chain along [010] has a zigzag topology with the backbone being defined by alternating eight-membered {PbONO}_2_ and {PbOCO}_2_ rings, Fig. 3.

### Experimental

1,10-Phenanthroline (1 mmol) was placed in one arm of a branched tube (Harrowfield *et al.*, 1996) and a mixture of lead(II) nitrate (1 mmol) and trichloroacetic acid (1 mmol) in the other. Methanol was then added to fill both arms, the tube sealed and the ligand-containing arm immersed in a bath at 333 K, while the other was left at ambient temperature. After two weeks, crystals had deposited in the arm held at ambient temperature. They were filtered off, washed with acetone and ether, and air-dried. Yield: 65%. *M*.pt. = 491 K.

### Refinement

Carbon-bound H-atoms were placed in calculated positions [C—H 0.95 Å, *U*_iso_(H) 1.2*U*_eq_(C)] and were included in the refinement in the riding model approximation.

### Crystal data

**Table:**

[Pb_2_(C_2_Cl_3_O_2_)_2_(NO_3_)_2_(C_12_H_8_N_2_)_2_]	*Z* = 1
*M**_r_* = 1223.54	*F*(000) = 572
Triclinic, *P*1	*D*_x_ = 2.415 Mg m^−^^3^
Hall symbol: -P 1	Mo *K*α radiation, λ = 0.71073 Å
*a* = 8.8852 (3) Å	Cell parameters from 9962 reflections
*b* = 9.7465 (3) Å	θ = 2.4–27.5°
*c* = 11.1570 (3) Å	µ = 10.54 mm^−^^1^
α = 109.427 (3)°	*T* = 100 K
β = 99.019 (3)°	Prism, colourless
γ = 106.199 (3)°	0.20 × 0.15 × 0.10 mm
*V* = 841.13 (4) Å^3^	

### Data collection

**Table:**

Agilent SuperNova Dual diffractometer with an Atlas detector	3899 independent reflections
Radiation source: SuperNova (Mo) X-ray Source	3740 reflections with *I* > 2σ(*I*)
Mirror monochromator	*R*_int_ = 0.029
Detector resolution: 10.4041 pixels mm^-1^	θ_max_ = 27.6°, θ_min_ = 2.4°
ω scan	*h* = −11→11
Absorption correction: multi-scan (*CrysAlis PRO*; Agilent, 2010)	*k* = −12→12
*T*_min_ = 0.227, *T*_max_ = 0.419	*l* = −14→14
12671 measured reflections	

### Refinement

**Table:**

Refinement on *F*^2^	Primary atom site location: structure-invariant direct methods
Least-squares matrix: full	Secondary atom site location: difference Fourier map
*R*[*F*^2^ > 2σ(*F*^2^)] = 0.017	Hydrogen site location: inferred from neighbouring sites
*wR*(*F*^2^) = 0.038	H-atom parameters constrained
*S* = 0.99	*w* = 1/[σ^2^(*F*_o_^2^) + (0.0188*P*)^2^] where *P* = (*F*_o_^2^ + 2*F*_c_^2^)/3
3899 reflections	(Δ/σ)_max_ = 0.001
235 parameters	Δρ_max_ = 0.84 e Å^−^^3^
0 restraints	Δρ_min_ = −0.97 e Å^−^^3^

### Fractional atomic coordinates and isotropic or equivalent isotropic displacement parameters (Å^2^)

**Table:**

	*x*	*y*	*z*	*U*_iso_*/*U*_eq_	
Pb	0.526354 (11)	0.321866 (10)	0.619700 (8)	0.00914 (4)	
Cl1	1.10668 (9)	0.71315 (9)	0.63924 (7)	0.02148 (16)	
Cl2	0.92529 (9)	0.85568 (8)	0.80608 (7)	0.01891 (15)	
Cl3	0.89253 (10)	0.85230 (9)	0.54481 (7)	0.02467 (17)	
O1	0.7927 (2)	0.5079 (2)	0.66123 (18)	0.0146 (4)	
O2	0.6722 (3)	0.5436 (2)	0.4883 (2)	0.0231 (5)	
O3	0.6734 (2)	0.1262 (2)	0.54516 (18)	0.0148 (4)	
O4	0.5454 (2)	0.1386 (2)	0.36793 (18)	0.0157 (4)	
O5	0.6385 (3)	−0.0491 (2)	0.35098 (19)	0.0167 (4)	
N1	0.5609 (3)	0.5650 (3)	0.8223 (2)	0.0112 (5)	
N2	0.6794 (3)	0.3366 (3)	0.8360 (2)	0.0115 (5)	
N3	0.6188 (3)	0.0712 (3)	0.4200 (2)	0.0115 (5)	
C1	0.5074 (3)	0.6760 (3)	0.8136 (3)	0.0131 (6)	
H1	0.4390	0.6586	0.7314	0.016*	
C2	0.5473 (4)	0.8182 (3)	0.9205 (3)	0.0163 (6)	
H2	0.5091	0.8964	0.9098	0.020*	
C3	0.6421 (4)	0.8433 (3)	1.0406 (3)	0.0152 (6)	
H3	0.6673	0.9378	1.1147	0.018*	
C4	0.7023 (3)	0.7275 (3)	1.0538 (3)	0.0115 (5)	
C5	0.6586 (3)	0.5890 (3)	0.9403 (3)	0.0111 (5)	
C6	0.7343 (3)	0.2258 (3)	0.8442 (3)	0.0120 (5)	
H6	0.7036	0.1326	0.7676	0.014*	
C7	0.8356 (4)	0.2399 (3)	0.9606 (3)	0.0154 (6)	
H7	0.8731	0.1580	0.9619	0.018*	
C8	0.8803 (3)	0.3727 (3)	1.0725 (3)	0.0150 (6)	
H8	0.9508	0.3849	1.1517	0.018*	
C9	0.8205 (3)	0.4908 (3)	1.0688 (3)	0.0113 (5)	
C10	0.7198 (3)	0.4687 (3)	0.9475 (2)	0.0098 (5)	
C11	0.8037 (4)	0.7453 (3)	1.1749 (3)	0.0151 (6)	
H11	0.8318	0.8382	1.2512	0.018*	
C12	0.8606 (3)	0.6330 (3)	1.1830 (3)	0.0147 (6)	
H12	0.9277	0.6478	1.2648	0.018*	
C13	0.7796 (3)	0.5824 (3)	0.5894 (3)	0.0123 (6)	
C14	0.9201 (3)	0.7446 (3)	0.6404 (3)	0.0142 (6)	

### Atomic displacement parameters (Å^2^)

**Table:**

	*U*^11^	*U*^22^	*U*^33^	*U*^12^	*U*^13^	*U*^23^
Pb	0.01030 (6)	0.00697 (6)	0.00857 (6)	0.00233 (4)	0.00028 (4)	0.00284 (4)
Cl1	0.0130 (3)	0.0269 (4)	0.0277 (4)	0.0043 (3)	0.0084 (3)	0.0157 (3)
Cl2	0.0239 (4)	0.0124 (3)	0.0149 (3)	0.0045 (3)	0.0009 (3)	0.0024 (3)
Cl3	0.0247 (4)	0.0209 (4)	0.0244 (4)	−0.0022 (3)	−0.0044 (3)	0.0178 (3)
O1	0.0157 (10)	0.0124 (10)	0.0149 (10)	0.0008 (8)	0.0043 (8)	0.0080 (8)
O2	0.0218 (12)	0.0128 (10)	0.0234 (11)	0.0013 (9)	−0.0097 (10)	0.0048 (9)
O3	0.0179 (11)	0.0173 (10)	0.0092 (9)	0.0073 (9)	0.0027 (8)	0.0047 (8)
O4	0.0187 (11)	0.0162 (10)	0.0154 (10)	0.0073 (9)	0.0038 (9)	0.0096 (8)
O5	0.0240 (11)	0.0096 (10)	0.0192 (10)	0.0078 (9)	0.0125 (9)	0.0043 (8)
N1	0.0115 (11)	0.0115 (11)	0.0106 (11)	0.0035 (9)	0.0022 (9)	0.0051 (9)
N2	0.0137 (12)	0.0088 (11)	0.0123 (11)	0.0039 (9)	0.0031 (10)	0.0046 (9)
N3	0.0120 (12)	0.0096 (11)	0.0135 (12)	0.0014 (9)	0.0063 (10)	0.0061 (9)
C1	0.0163 (14)	0.0149 (14)	0.0111 (13)	0.0092 (12)	0.0054 (11)	0.0053 (11)
C2	0.0230 (16)	0.0137 (14)	0.0165 (14)	0.0109 (12)	0.0077 (13)	0.0065 (11)
C3	0.0193 (15)	0.0109 (13)	0.0146 (14)	0.0046 (12)	0.0073 (12)	0.0033 (11)
C4	0.0130 (14)	0.0123 (13)	0.0107 (13)	0.0041 (11)	0.0055 (11)	0.0055 (10)
C5	0.0105 (13)	0.0116 (13)	0.0122 (13)	0.0013 (11)	0.0053 (11)	0.0069 (10)
C6	0.0144 (14)	0.0098 (13)	0.0111 (13)	0.0039 (11)	0.0027 (11)	0.0040 (10)
C7	0.0193 (15)	0.0144 (14)	0.0171 (14)	0.0081 (12)	0.0051 (12)	0.0101 (11)
C8	0.0170 (15)	0.0187 (15)	0.0131 (14)	0.0077 (12)	0.0035 (12)	0.0100 (11)
C9	0.0107 (13)	0.0125 (13)	0.0095 (13)	0.0025 (11)	0.0017 (11)	0.0047 (10)
C10	0.0096 (13)	0.0101 (13)	0.0101 (12)	0.0026 (10)	0.0041 (11)	0.0046 (10)
C11	0.0187 (15)	0.0121 (14)	0.0107 (13)	0.0036 (12)	0.0042 (12)	0.0015 (11)
C12	0.0125 (14)	0.0172 (14)	0.0095 (13)	0.0028 (11)	0.0003 (11)	0.0028 (11)
C13	0.0119 (14)	0.0110 (13)	0.0143 (14)	0.0045 (11)	0.0061 (12)	0.0038 (11)
C14	0.0124 (14)	0.0161 (14)	0.0145 (14)	0.0033 (11)	0.0018 (11)	0.0091 (11)

### Geometric parameters (Å, º)

**Table:**

Pb—O1	2.410 (2)	C1—H1	0.9500
Pb—O2^i^	2.821 (2)	C2—C3	1.371 (4)
Pb—O3	2.591 (2)	C2—H2	0.9500
Pb—O4	2.844 (2)	C3—C4	1.415 (4)
Pb—O5^ii^	2.807 (2)	C3—H3	0.9500
Pb—N1	2.576 (2)	C4—C5	1.412 (4)
Pb—N2	2.515 (2)	C4—C11	1.429 (4)
Cl1—C14	1.770 (3)	C5—C10	1.442 (4)
Cl2—C14	1.790 (3)	C6—C7	1.401 (4)
Cl3—C14	1.762 (3)	C6—H6	0.9500
O1—C13	1.260 (3)	C7—C8	1.369 (4)
O2—C13	1.226 (3)	C7—H7	0.9500
O3—N3	1.272 (3)	C8—C9	1.405 (4)
O4—N3	1.252 (3)	C8—H8	0.9500
O5—N3	1.247 (3)	C9—C10	1.413 (4)
N1—C1	1.322 (3)	C9—C12	1.442 (4)
N1—C5	1.366 (3)	C11—C12	1.350 (4)
N2—C6	1.326 (3)	C11—H11	0.9500
N2—C10	1.368 (3)	C12—H12	0.9500
C1—C2	1.401 (4)	C13—C14	1.568 (4)
			
O1—Pb—N2	77.20 (7)	C2—C3—C4	119.6 (2)
O1—Pb—N1	73.51 (7)	C2—C3—H3	120.2
N2—Pb—N1	65.43 (7)	C4—C3—H3	120.2
O1—Pb—O3	82.13 (6)	C5—C4—C3	117.2 (2)
N2—Pb—O3	77.41 (6)	C5—C4—C11	119.6 (2)
N1—Pb—O3	138.96 (6)	C3—C4—C11	123.2 (2)
O1—Pb—O5^ii^	143.59 (6)	N1—C5—C4	122.1 (2)
N2—Pb—O5^ii^	72.82 (7)	N1—C5—C10	118.4 (2)
N1—Pb—O5^ii^	110.82 (6)	C4—C5—C10	119.5 (2)
O3—Pb—O5^ii^	71.66 (6)	N2—C6—C7	123.0 (2)
O1—Pb—O2^i^	100.42 (6)	N2—C6—H6	118.5
N2—Pb—O2^i^	142.33 (6)	C7—C6—H6	118.5
N1—Pb—O2^i^	77.74 (6)	C8—C7—C6	119.4 (3)
O3—Pb—O2^i^	140.08 (6)	C8—C7—H7	120.3
O5^ii^—Pb—O2^i^	115.93 (6)	C6—C7—H7	120.3
O1—Pb—O4	90.38 (6)	C7—C8—C9	119.2 (2)
N2—Pb—O4	124.31 (6)	C7—C8—H8	120.4
N1—Pb—O4	159.41 (7)	C9—C8—H8	120.4
O3—Pb—O4	46.99 (5)	C8—C9—C10	118.1 (2)
O5^ii^—Pb—O4	89.76 (5)	C8—C9—C12	122.3 (2)
O2^i^—Pb—O4	93.09 (6)	C10—C9—C12	119.6 (2)
C13—O1—Pb	107.77 (17)	N2—C10—C9	121.8 (2)
N3—O3—Pb	101.84 (14)	N2—C10—C5	118.8 (2)
N3—O4—Pb	90.19 (14)	C9—C10—C5	119.3 (2)
C1—N1—C5	118.9 (2)	C12—C11—C4	121.5 (2)
C1—N1—Pb	123.02 (17)	C12—C11—H11	119.3
C5—N1—Pb	117.39 (16)	C4—C11—H11	119.3
C6—N2—C10	118.3 (2)	C11—C12—C9	120.6 (3)
C6—N2—Pb	122.22 (17)	C11—C12—H12	119.7
C10—N2—Pb	119.21 (16)	C9—C12—H12	119.7
O5—N3—O4	121.0 (2)	O2—C13—O1	128.1 (3)
O5—N3—O3	119.6 (2)	O2—C13—C14	118.2 (2)
O4—N3—O3	119.4 (2)	O1—C13—C14	113.8 (2)
N1—C1—C2	122.7 (2)	C13—C14—Cl3	113.04 (19)
N1—C1—H1	118.6	C13—C14—Cl1	108.31 (18)
C2—C1—H1	118.6	Cl3—C14—Cl1	109.62 (15)
C3—C2—C1	119.3 (3)	C13—C14—Cl2	108.31 (19)
C3—C2—H2	120.4	Cl3—C14—Cl2	107.89 (15)
C1—C2—H2	120.4	Cl1—C14—Cl2	109.62 (14)
			
N2—Pb—O1—C13	−162.22 (17)	C5—N1—C1—C2	0.3 (4)
N1—Pb—O1—C13	−94.40 (17)	Pb—N1—C1—C2	−170.2 (2)
O3—Pb—O1—C13	118.98 (17)	N1—C1—C2—C3	−1.8 (5)
O5^ii^—Pb—O1—C13	162.70 (14)	C1—C2—C3—C4	2.0 (4)
O2^i^—Pb—O1—C13	−20.64 (17)	C2—C3—C4—C5	−0.8 (4)
O4—Pb—O1—C13	72.57 (17)	C2—C3—C4—C11	179.0 (3)
O1—Pb—O3—N3	−105.10 (16)	C1—N1—C5—C4	1.0 (4)
N2—Pb—O3—N3	176.34 (16)	Pb—N1—C5—C4	172.0 (2)
N1—Pb—O3—N3	−158.57 (14)	C1—N1—C5—C10	−178.3 (2)
O5^ii^—Pb—O3—N3	100.50 (16)	Pb—N1—C5—C10	−7.3 (3)
O2^i^—Pb—O3—N3	−8.3 (2)	C3—C4—C5—N1	−0.7 (4)
O4—Pb—O3—N3	−7.15 (13)	C11—C4—C5—N1	179.4 (3)
O1—Pb—O4—N3	85.95 (15)	C3—C4—C5—C10	178.5 (2)
N2—Pb—O4—N3	11.24 (17)	C11—C4—C5—C10	−1.3 (4)
N1—Pb—O4—N3	123.84 (19)	C10—N2—C6—C7	−2.0 (4)
O3—Pb—O4—N3	7.11 (13)	Pb—N2—C6—C7	172.4 (2)
O5^ii^—Pb—O4—N3	−57.64 (15)	N2—C6—C7—C8	0.6 (4)
O2^i^—Pb—O4—N3	−173.59 (15)	C6—C7—C8—C9	1.3 (4)
O1—Pb—N1—C1	94.8 (2)	C7—C8—C9—C10	−1.8 (4)
N2—Pb—N1—C1	177.9 (2)	C7—C8—C9—C12	179.2 (3)
O3—Pb—N1—C1	150.9 (2)	C6—N2—C10—C9	1.5 (4)
O5^ii^—Pb—N1—C1	−123.5 (2)	Pb—N2—C10—C9	−173.1 (2)
O2^i^—Pb—N1—C1	−10.2 (2)	C6—N2—C10—C5	−179.0 (2)
O4—Pb—N1—C1	54.9 (3)	Pb—N2—C10—C5	6.4 (3)
O1—Pb—N1—C5	−75.88 (19)	C8—C9—C10—N2	0.4 (4)
N2—Pb—N1—C5	7.30 (18)	C12—C9—C10—N2	179.4 (2)
O3—Pb—N1—C5	−19.8 (2)	C8—C9—C10—C5	−179.1 (2)
O5^ii^—Pb—N1—C5	65.9 (2)	C12—C9—C10—C5	−0.1 (4)
O2^i^—Pb—N1—C5	179.2 (2)	N1—C5—C10—N2	0.7 (4)
O4—Pb—N1—C5	−115.7 (2)	C4—C5—C10—N2	−178.6 (2)
O1—Pb—N2—C6	−103.8 (2)	N1—C5—C10—C9	−179.7 (2)
N1—Pb—N2—C6	178.6 (2)	C4—C5—C10—C9	1.0 (4)
O3—Pb—N2—C6	−19.2 (2)	C5—C4—C11—C12	0.7 (4)
O5^ii^—Pb—N2—C6	55.2 (2)	C3—C4—C11—C12	−179.1 (3)
O2^i^—Pb—N2—C6	165.63 (18)	C4—C11—C12—C9	0.1 (4)
O4—Pb—N2—C6	−22.3 (2)	C8—C9—C12—C11	178.5 (3)
O1—Pb—N2—C10	70.51 (19)	C10—C9—C12—C11	−0.4 (4)
N1—Pb—N2—C10	−7.01 (18)	Pb—O1—C13—O2	−21.3 (3)
O3—Pb—N2—C10	155.2 (2)	Pb—O1—C13—C14	158.09 (17)
O5^ii^—Pb—N2—C10	−130.4 (2)	O2—C13—C14—Cl3	3.3 (3)
O2^i^—Pb—N2—C10	−20.0 (3)	O1—C13—C14—Cl3	−176.12 (19)
O4—Pb—N2—C10	152.07 (17)	O2—C13—C14—Cl1	−118.3 (2)
Pb—O4—N3—O5	167.5 (2)	O1—C13—C14—Cl1	62.2 (3)
Pb—O4—N3—O3	−12.2 (2)	O2—C13—C14—Cl2	122.8 (2)
Pb—O3—N3—O5	−165.96 (19)	O1—C13—C14—Cl2	−56.6 (3)
Pb—O3—N3—O4	13.7 (3)		

Symmetry codes: (i) −*x*+1, −*y*+1, −*z*+1; (ii) −*x*+1, −*y*, −*z*+1.
